# Effects of carbohydrate and protein supplement strategies on endurance capacity and muscle damage of endurance runners: A double blind, controlled crossover trial

**DOI:** 10.1080/15502783.2022.2131460

**Published:** 2022-10-12

**Authors:** Yiheng Liang, Yan Chen, Fan Yang, Jørgen Jensen, Ruirui Gao, Longyan Yi, Junqiang Qiu

**Affiliations:** a Department of Exercise Biochemistry, Exercise Science School, Beijing Sport University, Beijing, Peking, China; b Norwegian School of Sport Sciences, Department of Physical Performance, Oslo, Norway; c Institute of Sport and Health Science, Beijing Sport University, Beijing, Peking, China; d Beijing Sports Nutrition Engineering Research Center, Beijing Sport University, Beijing, Peking, China

**Keywords:** Endurance exercise, sports beverage, fatigue and recovery

## Abstract

**Background:**

The purpose of this study is to explore the effect of carbohydrate only or carbohydrate plus protein supplementation on endurance capacity and muscle damage.

**Methods:**

Ten recreationally active male runners (VO_2max_: 53.61 ± 3.86 ml/kg·min) completed run-to-exhaustion test three times with different intakes of intervention drinks. There was a 7-day wash-out period between tests. Each test started with 60 minutes of running at 70% VO_2max_ (phase 1), followed by an endurance capacity test: time-to-exhaustion running at 80% VO_2max_ (phase 2). Participants randomly ingested either 1) 0.4 g/kg BM carbohydrate before phase 1 and before phase 2 (CHO+CHO), 2) 0.4 g/kg BM protein before phase 1 and 0.4 g/kg BM carbohydrate before phase 2 (PRO+CHO), or 3) 0.4 g/kg BM carbohydrate before phase 1 and 0.4 g/kg BM protein before phase 2 (CHO+PRO). All subjects ingested carbohydrate (CHO) 1.2 g/kg BM during phase 1, and blood samples were obtained before, immediately, and 24 h after exercise for measurements of alanine aminotransferase (ALT), aspartate aminotransferase (AST), creatine kinase (CK), and myoglobin (MB).

**Results:**

There was no significant difference in time to exhaustion between the three supplement strategies (CHO+CHO: 432 ± 225 s; PRO+CHO: 463 ± 227 s; CHO+PRO: 461 ± 248 s). However, ALT and AST were significantly lower in PRO+CHO than in CHO+CHO 24 h after exercise (ALT: 16.80 ± 6.31 vs. 24.39 ± 2.54 U/L; AST: 24.06 ± 4.77 vs. 31.51 ± 7.53 U/L, *p* < 0.05). MB was significantly lower in PRO+CHO and CHO+PRO than in CHO+CHO 24 h after exercise (40.7 ± 15.2; 38.1 ± 14.3; 64.3 ± 28.9 ng/mL, respectively, *p* < 0.05). CK increased less in PRO+CHO compared to CHO+CHO 24 h after exercise (404.22 ± 75.31 VS. 642.33 ± 68.57 U/L, *p* < 0.05).

**Conclusion:**

Carbohydrate and protein supplement strategies can reduce muscle damage caused by endurance exercise, but they do not improve endurance exercise capacity.

## 1. Introduction

Supplementation in endurance runners generally aims to improve exercise performance and promote post-exercise recovery. Several studies have shown that carbohydrate and protein ingestion after exercise improves subsequent exercise performance better than intake of carbohydrate only [1–3]. Research has also reported that supplementing with extra protein during exercise can improve exercise performance compared to supplementing only carbohydrates during exercise [4–6]. These studies suggest that supplementation with additional protein during exercise improves performance, in part, due to a reduction in muscle damage [4,6,7]. However, other studies suggest that consuming carbohydrates plus protein during exercise did not improve exercise performance compared to carbohydrates alone under isocaloric conditions [8,9]. A study indicated no improvement in exercise performance when supplemented with lower carbohydrates plus protein compared with carbohydrates alone [10]. Conversely, under isocaloric conditions, some studies have suggested that adding protein to the recommended intake of carbohydrate beverages can improve exercise performance [1,11]. Furthermore, in a 1-week experiment, it was also suggested that carbohydrates plus protein can reduce muscle damage and improve performance compared to isocaloric carbohydrates [12]. Theoretically, the extra protein could speed up fuel absorption, as well as the greater insulin response, all of which affect the recovery of endurance performance [13,14]. Previous studies have studied the intake of supplements during or after exercise [1,4,6,11,12], and the effect of intake of supplements before exercise on performance deserves exploration.

Endurance exercise increases levels of cytokines and may increase markers of muscle, heart, liver, and kidney damage [15–17]. Creatine kinase (CK), myoglobin (MB), lactate dehydrogenase, and other indicators are commonly used as serum markers for exercise-induced muscle damage [18,19]. Alanine aminotransferase (ALT) and aspartate aminotransferase (AST) are considered liver enzymes, but they are also present in muscles, and increase in response to high-intensity exercise [18,20,21]. Previous studies have found that the markers of muscle damage increased after long-distance running [22,23]. It is worth noting that protein supplementation can reduce the exercise-induced elevation of these damage markers in the blood [6,24,25]. In the studies of carbohydrate and protein intake during exercise, it was also found that CK, LDH, and muscle soreness were significantly reduced after exercise after protein intake [4,7,9]. The reduction in muscle damage may be one reason for the improved endurance sports performance [4,6,7]. In general, ingesting 20–40 g of high-quality protein can increase muscle protein synthesis rate and reduce muscle damage [26–28]. However, consuming protein during long-distance exercise may increase protein oxidation, meaning that intake of protein may become fuel during exercise [29,30]. In order to understand the function of protein, we aim to explore whether protein supplementation before exercise can reduce muscle damage and improve performance. Moreover, in the study of Qin et al., it was pointed out that protein supplementation can enhance the sense of effort of exercise while reducing muscle pain and RPE [31].

Therefore, the main purpose of this study is to explore the effects of carbohydrate plus protein strategy on endurance capacity and muscle damage. We hypothesized that protein supplementation before exercise could better reduce muscle damage and improve performance compared to isocaloric carbohydrate supplementation alone.

## 2. Materials and methods

### Participants

Eleven recreationally active male runners who trained at least three times a week (over 6 h) were recruited from China Athletics College of Beijing Sports University. Participants were forbidden from taking other sports supplements during the experiment, and were asked to avoid competition and high-intensity training for 3 days prior to testing. During the experiment, one subject could not complete subsequent experiments due to injury. Therefore, a total of 10 subjects completed the entire experimental procedure (age: 21 ± 2; height: 175.1 ± 4.2 cm; weight: 62.8 ± 5.3 kg; VO_2max_: 53.61 ± 3.86 ml/kg·min). Before the experiment, subjects were informed of the experimental procedures and possible risks and asked to sign an informed consent. All procedures of the experiments were approved by the Institutional Review Board of Beijing Sport University (BSU IRB; 2019110H).

### Experimental design

The study had a randomized, crossover, double-blind design. Participants performed three run-to-exhaustion tests and were assigned different supplements in a random order in each running test. There was a 7-day washout period between each test.

**Figure 1.**
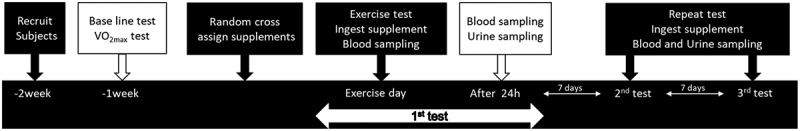
Experimental design. Obtained venous blood before the test, immediately after the test, and 24 h after the test. Morning urine collected 24 h after the test.

Thirty minutes before the test, the subjects ingested the assigned pre-running supplement. Before starting the test, a venous blood sample was taken and muscle soreness was recorded by the Visual Analog Scale (VAS). The VAS is a Likert-based scale used to identify the severity of muscle soreness on a 10-point scale, with “0” indicating no soreness and “10” indicating extreme soreness [32–34]. After running to exhaustion test, the subject’s venous blood was collected, and muscle soreness was recorded by VAS. The morning urine was collected on the second day of the exercise test, and venous blood was collected and the severity of muscle soreness recorded 24 h after the end of the test (Figure 1).

### Experimental running protocol

#### Preliminary test

The preliminary test included a venous blood, morning urine collection, and the maximum oxygen uptake (VO_2max_) was tested. We tested VO_2max_ according to the previous literature [35] and calculated the corresponding speed at different VO_2max_ intensities. VO_2max_ tests were performed on the running treadmill (h/p/cosmos Mercury 4.0, Germany) using the gas metabolism test system (COSMED k4b2, Italy). All subjects were asked not to perform any heavy exercise for the first 3 days of the preliminary test and to maintain a normal diet and sleep time.

#### Run-to-exhaustion test

**Figure 2.**
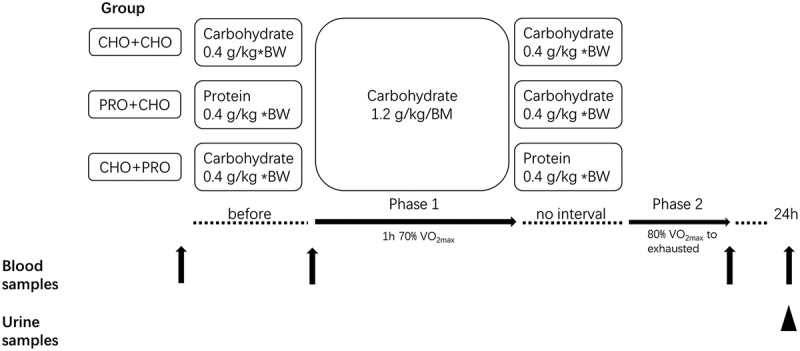
Blood and urine collection, ingredients of supplements. Black arrows represent blood collection; black triangles represent urine collection.

The run-to-exhaustion tests were performed on an indoor running treadmill (h/p/cosmos Mercury 4.0, Germany). The run-to-exhaustion test was divided into two phases with no time interval between the two phases. In phase 1, the subjects ran for 60 minutes at 70% VO_2max_ on the running treadmill with a slope of 0°. After completing the 60-minute running, the subjects ran to exhaustion at 80% VO_2max_ with a slope of 0° in the phase 2. In phase 2, if the subject proposes to stop the test, or if there is an abnormal physiological state such as difficulty breathing, chest discomfort, dizziness, and so on, the test will be stopped. Consistent verbal encouragement was given to all subjects by the same investigators during each test phase 2 to bring the subjects to true exhaustion (Figure 2).

The subjects were supplemented with three different supplement strategies in random order for the three running tests. The three different supplement strategies were 1) ingested carbohydrate at 30 minutes before phase 1 and before phase 2 (CHO+CHO); 2) ingested protein at 30 minutes before phase 1 and ingest carbohydrate before phase 2 (PRO+CHO); and 3) ingested carbohydrate at 30 minutes before phase 1 and ingest protein before phase 2 (CHO+PRO).

#### Solution composition

The three supplement strategies had the same caloric intake. The protein in the supplement was whey protein isolate (whey protein isolate LacprodanDI-9224, Arla Foods Ingredients P/S) and each supplement dose was 0.4 g/kg BM. The carbohydrate was maltodextrin (Xiwang Group, China), and each supplement dose was 0.4 g/kg BM. At the same time, the subjects were supplemented with carbohydrate at 1.2 g/kg BM during phase 1, which was ingested every 10 minutes in the form of a liquid. All supplements were formulated with water at a concentration of 20% and contained artificially sweetened solution (no calorie) to simulate the same taste. Subjects ingested supplements from the same opaque bottles for each test to make it impossible for them to distinguish supplements by appearance, and subjects were unable to distinguish between supplements by taste because of the artificially sweetened solution. The distribution of supplements was done by staff unrelated to the study. The experimenters and the subject were blinded to the group of supplements the subject received for each test (Figure 2).

#### Dietary control

The subjects were required to adhere to the experimental dietary recommendations (the energy intake proportion of the three main nutrients is 50% carbohydrates, 20% proteins, and 30% fats). Subjects recorded their daily calorie intake 1 day before the test, on the day of the test, and the day after the end of the test on their mobile phone application (Boohee, Information Technology Co., Ltd., Shanghai, China). We asked subjects to be consistent in their food intake over the 3 days of each test to reduce the effect of diet on the experimental results (Table 1).

**Table 1:** Subjects total dietary intake.

	Before the test day	Test day	After the test day
Energy, kcal	2625.40 ± 308.64	2633.00 ± 257.44	2430.90 ± 254.01
CHO, g	340 ± 36.06	346.1 ± 31.17	310.6 ± 24.45
Protein, g	100.8 ± 16.4	100.2 ± 13.1	105.2 ± 16.97
Fat, g	95.8 ± 13.95	94.2 ± 16.46	85.3 ± 12.83

#### Blood and urine analysis

A baseline venous blood sample was collected from the subjects before the experiment, and in the next three run tests, the venous blood was obtained before each test, immediately after the test, and 24 h after the test (Figure 2). Blood samples were collected into a 5 ml gel serum tube (Vacuette, Frickenhausen, Germany). Alanine aminotransferase (ALT), aspartate aminotransferase (AST), and creatine kinase (CK) were determined with a fully automatic biochemical analyzer (Beckman DXI 800, Beckman Coulter, Fullerton, CA, USA). Myoglobin (MB), insulin, testosterone (T), and cortisol (C) were measured with a fully automatic immuno-analyzer (Beckman DXC 800, Beckman Coulter, Fullerton, CA, USA).

Before the start of the experiment, the subjects collected morning urine as a baseline value. For 3 days before and after the baseline morning urine collection, heavy exercise was forbidden, and energy intake was based on the experimentally recommended diet. In the three subsequent tests, morning urine was collected the next day after the test (Figure 2). 3-Methylhistidine (3-MH) in urine was determined with ELISA (BOS-47007, BOSK, China).

## 3. Statistical analysis

We planned to detect a moderate effect size (0.4ES) on primary outcome (muscle damage; CK, MB), with a statistical power of 80% and α level (2-tailed) of 5% for a repeated-measure design, resulting in a minimum of six subjects (calculated by G*Power 3.1.9.2; software developed at Dusseldorf University, Germany). In order to support within-study participants’ drop-outs or exclusions, the experiment eventually recruited 11 subjects. SPSS 20.0 was used to analyze all the data in the experiment, and the experimental data were expressed as mean ± standard deviation. Repeated measurements *ANOVA* was used to perform statistical analysis on AST, ALT, insulin, CK, T, C, and VAS to compare these indicators at different timings (before the test, immediately after the test, and 24 h after the test) and the differences between the three supplement strategies, and Bonferroni was used as a post hoc test. Exhaustion time and 3-MH were performed through a one-way analysis of variance. When *p* < 0.05, it is defined as a significant difference in data.

## 4. Results

### Endurance capacity

There was no significant difference in time-to-exhaustion running at 80% of VO_2max_ after running for 60 minutes at 70% VO_2max_ between the three supplement strategies. Times to exhaustion of the three interventions were CHO+CHO: 432 ± 225 s; PRO+CHO: 463 ± 227 s; and CHO+PRO: 461 ± 248 s.

### Blood and urine analyses

Insulin concentration increased significantly above baseline value after intake of all three different supplement strategies. Immediately after exercise, insulin concentrations of the three supplementation strategies were significantly lower compared to before exercise (Figure 4a). In PRO+CHO, immediately after exercise, plasma insulin was still significantly higher than the baseline value. However, there was no significant difference in insulin concentration before and immediately after exercise between the three supplement strategies.

The CK values after exercise with the three supplementation strategies increased significantly. At only 24 h after exercise, the CK value of PRO+CHO group was significantly lower than that of CHO+CHO (413.40 ± 76.70 vs 632.30 ± 72.01 U/L, *p* = 0.026) (Figure 4b). MB values of the three dietary supplement strategies increased immediately after exercise, but there was no significant difference between the three supplement strategies. At 24 h after exercise, the MB values in PRO+CHO and CHO+PRO groups were significantly lower than those in CHO+CHO (PRO+CHO: 40.7 ± 15.2 ng/mL; CHO+PRO: 38.1 ± 14.3 ng/mL; CHO+CHO: 64.3 ± 28.9 ng/mL; *p* = 0.047; *p* = 0.025, respectively) (Figure 4c).

ALT and AST values immediately after exercise increased with all three dietary supplement strategies. ALT and AST in PRO+CHO group were significantly lower than in CHO+CHO group immediately after exercise (ALT: 22.75 ± 6.22 vs 31.74 ± 4.79 U/L, *p* = 0.005; AST: 23.14 ± 3.30 vs 29.39 ± 5.16 U/L, *p* = 0.012, respectively). At 24 h after exercise, AST and ALT in PRO+CHO group were also significantly lower than in CHO+CHO group (ALT: 16.80 ± 6.31 vs 24.39 ± 2.54 U/L, *p* = 0.023; AST: 24.06 ± 4.77 vs 31.51 ± 7.53 U/L, *p* = 0.04, respectively) (Figure 4 d, e).

Immediately after exercise, the T and C values were significantly higher than before the exercise. Twenty-four hours after exercise, the value of C was significantly lower than immediately after exercise. T values also decreased 24 h after exercise compared to immediately after exercise, but there was no significance. However, there were no significant differences in T and C values in different treatments.

**Figure 3.**
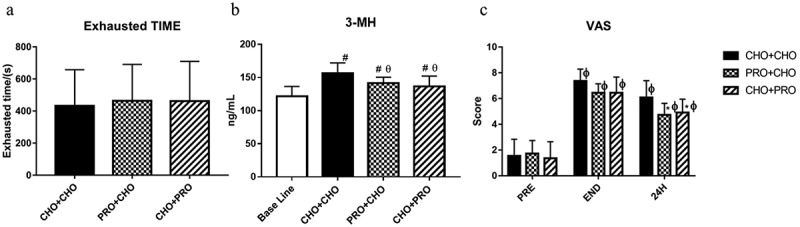
Exhaustion time, 3-MH, and VAS of different supplement strategies. 3-MH was collected the next day after the test. Immediately after exercise (END). Twenty-four hours after exercise (24 H). a. Time to exhaustion was achieved by running at 80% VO_2max_, following 60 minutes at 70% VO_2max_. b. # indicates a significant difference from the baseline (*p* < 0.05). θ indicates a significant difference from the CHO+CHO group (*p* < 0.05). c. * indicates a significant difference compared with CHO+CHO 24 h after exercise (*p* < 0.05). φ indicates a significant difference compared with the value of before exercise (*p* < 0.05).

3-MH was significantly higher 24 h after exercise compared to baseline (baseline: 124.53 ± 16.25 ng/mL; PRO+CHO: 141.74 ± 8.35 ng/mL, *p* = 0.007; CHO+PRO: 138.74 ± 15.98 ng/mL, *p* = 0.025; CHO+CHO: 156.73 ± 15.10 ng/mL, *p* = 0.001, respectively). Twenty-four hours after exercise, 3-MH in PRO+CHO and CHO+PRO groups was significantly lower than in CHO+CHO group (*p* = 0.018; *p* = 0.05, respectively) (Figure 3b).

**Figure 4.**
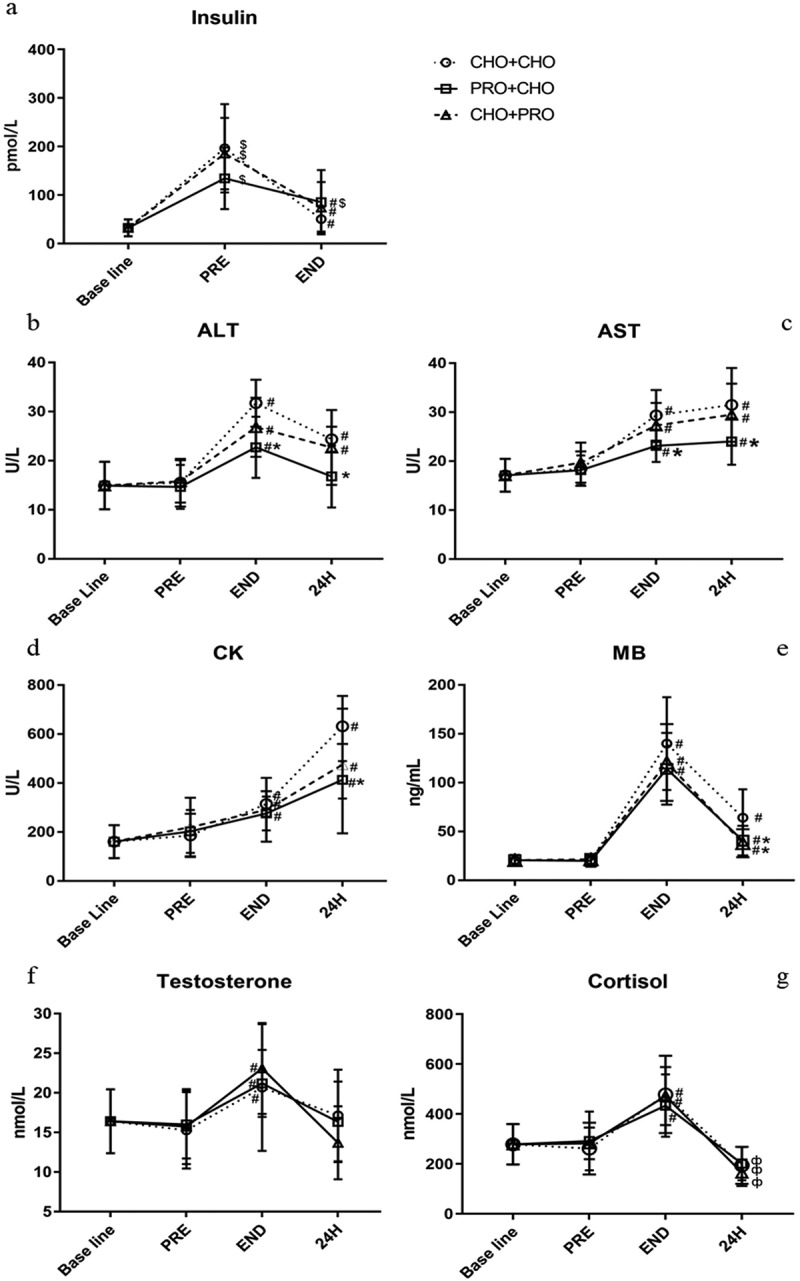
Biochemical indicators in the blood. Before the exercise (PRE). Immediately after exercise (END). Twenty-four hours after exercise (24 H). Alanine aminotransferase (ALT), aspartate aminotransferase (AST), creatine kinase (CK), myoglobin (MB), testosterone (T), and cortisol (C). The circle indicates CHO+CHO; square indicates PRO+CHO; triangle indicates CHO+PRO. * indicates a significant difference compared with CHO+CHO (*p* < 0.05). $ indicates a significant difference compared with baseline. # indicates a significant difference compared with before exercise (*p* < 0.05). ф indicates a significant difference compared with immediately after exercise.

Compared with before exercise, the VAS values of the three supplement strategies significantly increased immediately after exercise and 24 h after exercise. There was no significant difference in VAS values between the three dietary supplement strategies immediately after exercise, but 24 h after exercise, the VAS of PRO+CHO and CHO+PRO groups was significantly lower than that of CHO+CHO group (*p* = 0.017; *p* = 0.028, respectively).

## 5. Discussion

### Endurance capacity

In the present study, time to exhaustion at 80% VO_2max_ did not differ between the three isocaloric supplement strategies and protein intake did not improve endurance capacity. However, intake of protein before and during exercise reduced several markers of muscle damage compared to intake of carbohydrate only.

Saunders et al. [7] found that supplementing carbohydrates with additional protein improved exercise performance. However, it is hard to ignore the effect of proteins providing extra calories on exercise performance. Other studies where supplemental calories were equivalent have not shown improved endurance performance when protein was supplemented with carbohydrate before or during the endurance performance test [8,9,36,37]. In our study, the three supplement strategies had the same caloric intake, which may be the reason why we did not find improved performance as previous studies did [5,7,10]. The other reason may be related to exercise intensity. One study suggested that the relative exercise intensity of an individual may be a factor in determining the efficacy of carbohydrate and carbohydrate-plus-protein supplements [38]. Ferguson et al. [10] suggested that compared to carbohydrate supplementation alone, carbohydrate plus protein can improve aerobic endurance during exercise intensity near the ventilatory threshold. They suggested that high-intensity exercise leads to other fatigue factors, such as a significant decrease in muscle pH or depletion of high-energy phosphates, which supplements may not affect. In this experiment, the subjects exercised to exhaustion at 80% VO_2max_, which may be one reason why we did not find differences in endurance capacity.

In the present study, there was no significant difference in insulin concentration between the three supplement strategies before and immediately after exercise. This may be related to the amount and type of carbohydrate supplement in this experiment. The amount of carbohydrates we provided in the experiment was higher than that recommended in the American College of Sports Medicine Position Statement [39] because we wanted protein to reduce muscle damage rather than work as fuel when energy is insufficient [29,30]. Perhaps because we provided sufficient carbohydrates, there was no difference in exhausted time or insulin among the three strategies. Compared with the mixed carbohydrate (dextrose, fructose, maltodextrin) consumed in other experiments [4,6,10,36], the carbohydrate use in this study was single maltodextrin, which may also be one of the reasons affecting insulin and exhaustion time. In this experiment, the supplementation of carbohydrate and protein was performed separately. However, in those experiments that found the combination of carbohydrate and protein supplementation can enhance the insulin response, carbohydrate and protein were ingested together [6,10,36]. This may be the reason why we did not find a difference in insulin concentrations between the three supplement strategies.

### Muscle damage and fatigue recovery

In this experiment, ALT and AST values of the PRO+CHO group were significantly lower than in CHO+CHO group immediately and 24 h after exercise. This might be explained by the fact that extra protein provides more amino acids for the muscle, which reduce muscle cell damage caused by repeated muscle contraction during long-distance running as previously reported after eccentric exercise [6,25,31]. On the other hand, the ALT and AST values of the CHO+PRO group were also lower than those of CHO+CHO group, but there was no statistical difference. It seems that protein supplementation is a better way than carbohydrate supplementation alone to reduce muscle damage caused by running and accelerate recovery after exercise, and protein supplementation before exercise has a better protective effect on the damage caused by long-term running.

Although protein is not the main energy source in exercise, it still contributes to total energy consumption during prolonged aerobic or endurance exercise [26,40]. One hypothesis in the previous study was that the reduction of free amino acid pool in muscle tissue may cause muscle protein breakdown [41]. In this study, MB was significantly lower 24 h after exercise when protein was ingested compared to CHO+CHO. In addition, only PRO+CHO group’s CK was significantly lower than that of CHO+CHO. Romano et al. [9] also found that although carbohydrate and protein supplementation did not prolong fatigue time under isocaloric conditions, it reduced exercise-induced CK and lactate dehydrogenase. In studies of exercise performance improved by additional protein supplementation, additional protein reduced CK and MB and alleviated exercise-induced muscle damage [4,6,7,42]. The reason protein supplement during exercise was effective might be that the protein intake in advance increases the concentration of amino acids in the blood, thereby reducing muscle damage and speeding up recovery after exercise. Moreover, previous studies have shown that prolonged exercise can increase intaking protein oxidation [29,30]. In this experiment, we provided 1.2 g/kg BM carbohydrates during exercise in order to make the intaking protein better perform its function rather than serving as fuel during exercise. This may explain why there was no significant difference in muscle damage between the protein supplementation groups. Furthermore, in the two groups supplemented with protein, the VAS at 24 h after exercise was lower than that of CHO+CHO group. This may be partly because the supplemented protein increased the recovery time of skeletal muscle. On the other hand, the intake of protein may increase central neuronal neurotransmitters, such as serotonin [43], thereby reducing VAS levels.

This experiment also tested the indicators of muscle degradation in urine and found that the level of 3-MH in urine in the three supplement strategies after exercise increased significantly compared with baseline. After exercise, the 3-MH level in PRO+CHO and CHO+PRO groups was significantly lower than that of CHO+CHO group, but there was no significant difference between PRO+CHO and CHO+PRO groups. 3-MH can reflect the degradation of skeletal muscle protein due to muscle contraction during exercise [44]. The two groups supplemented with protein in this experiment were significantly lower than the CHO+CHO group. A possible explanation for this is that the protein supplement provided more amino acids during exercise than in the carbohydrate-only group, thereby resulting in attenuation of increases in 3-MH and speeding up recovery after exercise. However, in this experiment, we did not maintain that the subjects were always in a constant standard state of hydration and lacked the measurement of the urine-specific gravity of the subjects’ morning urine to determine the concentration of urine. This may also be responsible for changes in 3-MH in the urine.

The experimental data showed that there was no significant difference in T and C in the three supplement strategies at different time points. As the most active androgen in the body, testosterone can stimulate tissues to take up amino acids and promote protein synthesis [45,46]. Overall, testosterone can promote anabolic metabolism in the body. A study by Beelen et al. [47] showed that although continuous endurance exercise stimulates muscle protein synthesis, protein supplementation during continuous endurance exercise did not increase the rate of muscle protein synthesis. Our experiment did not measure protein synthesis indicators, but the experimental insulin results also did not appear significantly different among the three supplement strategies. Another reason that the testosterone did not appear to be different may be due to insufficient doses of protein supplements, especially leucine. Pasiakos et al. [48] observed that the measurement of leucine will significantly affect muscle protein synthesis. Although there was no significant difference in T and C between the three supplement strategies in this experiment, it can be found from the results described above that the muscle damage indicator of the two groups supplemented with protein was significantly lower than the carbohydrate-only group. Protein supplementation increased the concentration of amino acids in the body, thereby reducing muscle damage caused by endurance exercise and speeding up recovery after exercise. However, the protein supplement dosage may not significantly change the muscle protein synthesis. Therefore, the data showed that the muscle damage indicator of the two groups supplemented with protein was lower than that of the carbohydrate-only group, but there was no difference in T and C between the three supplement strategies.

## 6. Limitation

The subjects we recruited were recreationally active male runners, and the experiment was conducted by running. But future research could explore different gender and age groups, as well as other forms of exercise. In addition, we used a singular CHO source (maltodextrin), and the mixed CHO may better promote glycogen synthesis to improve exercise performance. Unfortunately, we did not measure blood glucose and blood lactate, so glycogen synthesis and the mechanism of exhaustion is not clear. We did not measure the urine-specific gravity of the subjects to ensure hydration, which should be noted in future experiments. This experiment is a short-term acute experiment; however, there are uncertainties about long-term adaptive changes, especially exercise performance, muscle mass, and body composition, and it is worth continuing to explore.

## 7. Conclusion

In the case of sufficient carbohydrate intake, protein intake before or during exercise cannot extend the time that runners run to exhaustion in endurance exercise. For endurance exercise, compared with carbohydrate supplementation alone, the supplement of carbohydrates and protein strategy can effectively reduce muscle damage caused by endurance exercise and promote fatigue recovery after exercise.

## Data Availability

The datasets generated and/or analyzed as part of the current study are not publicly available due to confidentiality agreements with subjects. However, they can be made available solely for the purpose of review and not for the purpose of publication from the corresponding author upon reasonable request.

## Abbreviations

CHO carbohydrate

PRO protein

ALT alanine aminotransferase

AST aspartate aminotransferase

CK creatine kinase

MB myoglobin

T testosterone

C cortisol

3-MH 3-methylhistidine.

## Author details

^a^Department of Exercise Biochemistry, Exercise Science School, Beijing Sport University, No. 48 Xinxi Road, Haidian District, Beijing, China. ^b^Institute of Sport and Health Science, Beijing Sport University, No. 48 Xinxi Road, Haidian District, Beijing, China. ^c^Department of Physical Performance, Norwegian School of Sport Sciences, Sognsveien 220 Ullevål Stadion, Oslo, Norway. ^d^Beijing Sports Nutrition Engineering Research Center, Beijing Sport University, No. 48 Xinxi Road, Haidian District, Beijing, China.
